# Isolating promoters from *Corynebacterium ammoniagenes* ATCC 6871 and application in CoA synthesis

**DOI:** 10.1186/s12896-019-0568-9

**Published:** 2019-11-12

**Authors:** Yingshuo Hou, Siyu Chen, Jianjun Wang, Guizhen Liu, Sheng Wu, Yong Tao

**Affiliations:** 1 CAS Key Laboratory of Microbial Physiological and Metabolic Engineering, Institute of Microbiology, Chinese Academy of Sciences, Beijing, 100101 People’s Republic of China; 2 University of Chinese Academy of Sciences, Beijing, 100101 People’s Republic of China; 3 Kaiping Genuine Biochemical Pharmaceutical Co. Ltd, Kaiping, People’s Republic of China

**Keywords:** *Corynebacterium ammoniagenes*, Transcriptome, Promoter, Coenzyme A

## Abstract

**Background:**

*Corynebacterium ammoniagenes* is an important industrial organism that is widely used to produce nucleotides and the potential for industrial production of coenzyme A by *C. ammoniagenes* ATCC 6871 has been shown. However, the yield of coenzyme A needs to be improved, and the available constitutive promoters are rather limited in this strain.

**Results:**

In this study, 20 putative DNA promoters derived from genes with high transcription levels and 6 promoters from molecular chaperone genes were identified. To evaluate the activity of each promoter, red fluorescence protein (RFP) was used as a reporter. We successfully isolated a range of promoters with different activity levels, and among these a fragment derived from the upstream sequence of the 50S ribosomal protein L21 (P_*rpl21*_) exhibited the strongest activity among the 26 identified promoters. Furthermore, type III pantothenate kinase from *Pseudomonas putida* (*Pp*coaA) was overexpressed in *C. ammoniagenes* under the control of P_*rpl21*_, CoA yield increased approximately 4.4 times.

**Conclusions:**

This study provides a paradigm for rational isolation of promoters with different activities and their application in metabolic engineering. These promoters will enrich the available promoter toolkit for *C. ammoniagenes* and should be valuable in current platforms for metabolic engineering and synthetic biology for the optimization of pathways to extend the product spectrum or improve the productivity in *C. ammoniagenes* ATCC 6871 for industrial applications.

## Background

*Corynebacterium ammoniagenes* (formerly known as *Brevibacterium ammoniagenes*) is a Gram-positive, non-pathogenic soil bacterium with high guanine–cytosine (GC) DNA content. Due to the divergent evolution of *C. ammoniagenes*, different strains are used to produce different metabolites. For example, *C. ammoniagenes* ATCC 6872 is used in the production of nucleotides and nucleosides [1], while *C. ammoniagenes* ATCC 6871 shows a relatively high synthesis capacity of coenzyme A (CoA), which is a ubiquitous and essential cofactor found in all three domains of life and is involved in numerous metabolic pathways [2–4].

Sequencing of the *C. ammoniagenes* genome has helped to identify key enzymes for diverting carbon flow from metabolic pathways to other products [5, 6]. However, a major breakthrough in genetic manipulation to precisely regulate the expression of individual biosynthetic genes is still in process. Progress in the manipulation of *C. ammoniagenes* genes has been achieved through the development of effective transformation protocols and cloning vectors [7]. Most vectors are based on the related *C. glutamicum* endogenous cryptic plasmids that have incorporated *E. coli* elements for transfer between the species [8–10]. Almost all these vectors have adopted *E. coli* promoters or *C. ammoniagenes* native promoters to express foreign proteins in *C. ammoniagenes* [11–13]. Although some of these promoters are active in *C. ammoniagenes*, the activities are quite low [14].

To efficiently engineer *C. ammoniagenes*, it is necessary to screen endogenous promoters. Paik and coworkers isolated 22 representative promoters from the genome of *C. ammoniagenes* ATCC 6872 and the activity of overexpressed chloramphenicol transacetylase from the strongest promoter IJ73 was 2.85 U [7]. Similar studies have been performed by Park and coworkers, where they overexpressed the attenuator binding protein in *C. ammoniagenes* ATCC 6872 using the endogenous promoter CJ1 [15]. However, the above promoters have not been universally applied in *C. ammoniagenes* ATCC 6871 (see text), probably due to the divergent evolution of *C. ammoniagenes* genomes. For instance, the sequence identities of promoters IJ59 and IJ73 from *C. ammoniagenes* ATCC 6872 with the homologous promoters from *C. ammoniagenes* ATCC 6871 are 93 and 89%, respectively. Therefore, due to the importance of CoA, it is necessary to find promoters that are effective in *C. ammoniagenes* ATCC 6871.

Endogenous promoters can be obtained by genomic dissection or promoter trapping et.al [16]. Nonetheless, with the advent of genomics and transcriptomics, we can isolate promoters more rationally. Studies have shown that strong promoters are usually obtained from the promoters of essential genes whose transcription levels are presumed to be high and constant [17]. In addition, we found that some commonly-used strong promoters such as P_*dnak*_ in *Gluconobacter oxydans* and P_*gro*_ in *Corynebacterium glutamicum* are promoters of molecular chaperones [18, 19]. In this study, the promoters for 20 genes with high transcription levels and 6 molecular chaperone-encoding genes are identified by bioinformatic methods and their activities were further examined experimentally. Among them, P_*rpl21*_ was found to be the strongest promoter, which was used in the biosynthesis of CoA, increasing the yield by 4.4 times.

## Results

### Screening promoters and construction of probe-vector

**Fig. 1:**
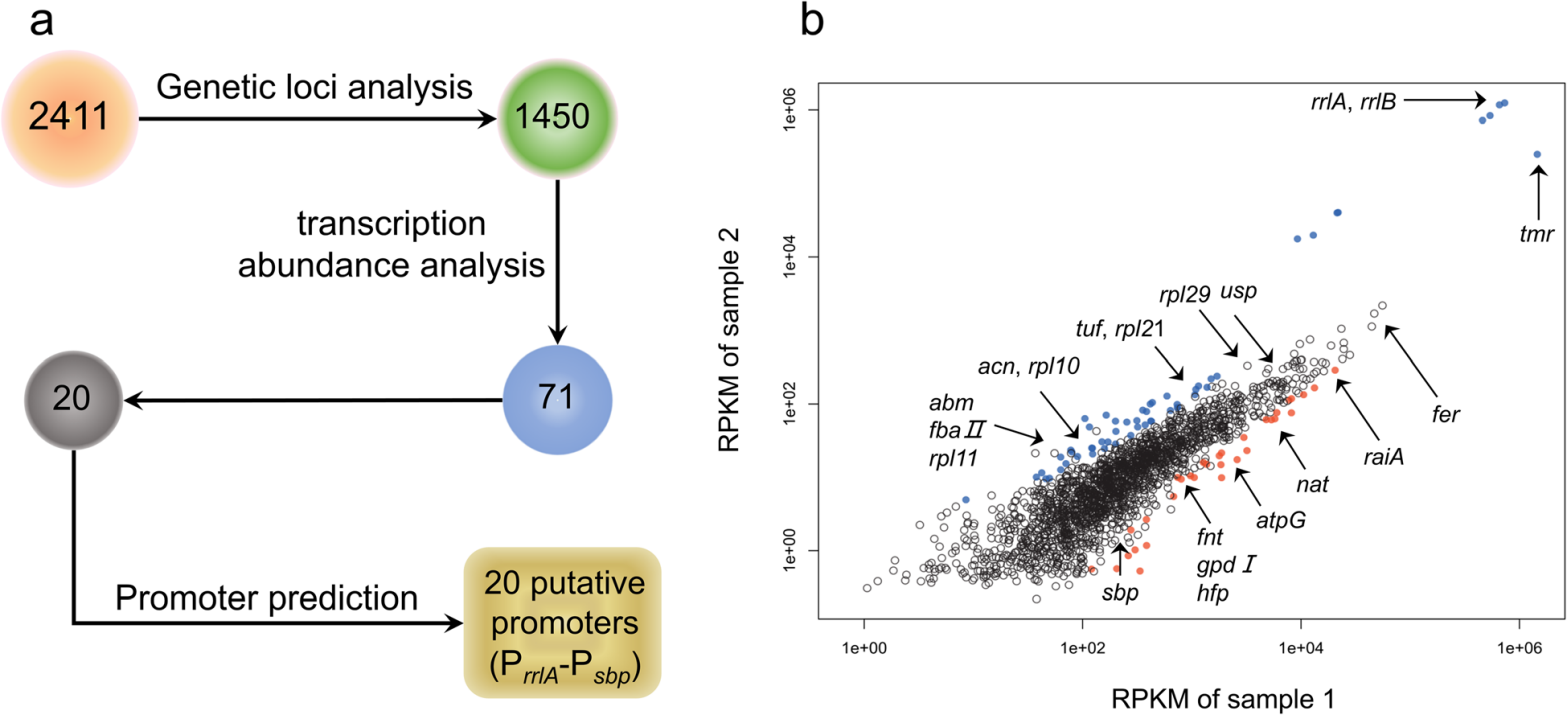
Rational selection of promoters form RNA-seq profile. **a**: The flowchart for selection of promoters from the transcriptomic data. **b**: The transcription levels of selected 20 genes in two different samples

*C. ammoniagenes* ATCC 6871 cells grown in LB medium and fermentation medium to the exponential phase (OD_600nm_ ≈ 2.0) were collected and sent to Mega genomics (Beijing, China) for RNA-seq. A total of 2411 genes were identified from the transcriptomic data. Considering that there are many cases in which genes are in the same operon, genetic loci analysis was performed and the results showed that 1508 genes in 547 operons were expressed. In general, genes in the same operons share a common promoter, so the first gene in each operon was selected. Thus, the total number of remaining genes was 1450 (2411–1508 + 547). Transcription abundance analysis was used to select 71 genes with transcription levels in the top 100 in both RNA-seq profiles. Sequences upstream of the top 20 genes were predicted and scored using the online promoter prediction tool as described below. The annotations and RPKM values for these genes are shown in Table 1. A transcriptomic data analysis flow chart and the transcription levels of genes are shown in Fig. 1.

**Table 1:** Identified molecular chaperone genes or those with high level expression from RNA-seq data

Name	Downstream Product	Average RPKM	Score
P_*rrlA*_	23S ribosomal RNA	988,873	0.99
P_*rrlB*_	23S ribosomal RNA	913,696	0.97
P_*tmr*_	Transfer-messenger RNA	853,640	0.96
P_*fer*_	Ferritin	46,832	0.92
P_*raiA*_	Ribosomal subunit interface protein	44,446	0.91
P_*usp*_	Universal stress protein	28,915	0.83
P_*rpl29*_	50S ribosomal protein L29	24,596	0.89
P_*nat*_	N-acetyltransferase	18,830	0.96
P_*atpG*_	F0F1 ATP synthase subunit gamma	16,820	0.80
P_*tuf*_	Elongation factor Tu	14,228	0.98
P_*rpl21*_	50S ribosomal protein L21	13,153	0.81
P_*fnt*_	NarK/NasA family nitrate transporter	12,650	0.94
P_*gpdI*_	Type I glyceraldehyde-3-phosphate dehydrogenase	12,223	0.83
P_*hfp*_	Hemoglobin flavoprotein	12,078	0.86
P_*acn*_	Aconitate hydratase	10,439	0.84
P_*rpl10*_	50S ribosomal protein L10	10,398	1.00
P_*abm*_	Antibiotic biosynthesis monooxygenase	9935	0.73
P_*fbaII*_	Class II fructose-bisphosphate aldolase	9740	0.77
P_*rpl11*_	50S ribosomal protein L11	9473	0.82
P_*sbp*_	ABC transpoter, substrate-binding protein	9120	0.99
P_*groELA*_	Molecular chaperone GroEL	6594	0.89
P_*groELB*_	Molecular chaperone GroEL	6261	0.87
P_*dnaJA*_	Molecular chaperone DnaJ	239	0.85
P_*dnaJB*_	Molecular chaperone DnaJ	703	0.87
P_*groES*_	Co-chaperone GroES	2215	1.00
P_*dnaK*_	Molecular chaperone DnaK	4523	0.98

Previous studies have shown that most promoters of molecular chaperones exhibit high transcription levels in prokaryotes, so all 6 annotated molecular chaperones in the *C. ammoniagenes* ATCC 6871 genome (Accession Number: NZ_CP009244) (two GroEL, two DnaJ, one DnaK, and one Co-chaperone GroES) were identified and their promoters were predicted as described in materials and methods. The information of these genes and transcriptomic RPKM values are also listed in Table 1. By predicting the promoter regions, 26 promoters were constructed according to the rules described below and their corresponding RFP expression plasmids were named as pXMJ190-P_*n*_. The sequences of the 26 promoters are listed in Additional file 1: Table S3.

**Fig. 2:**
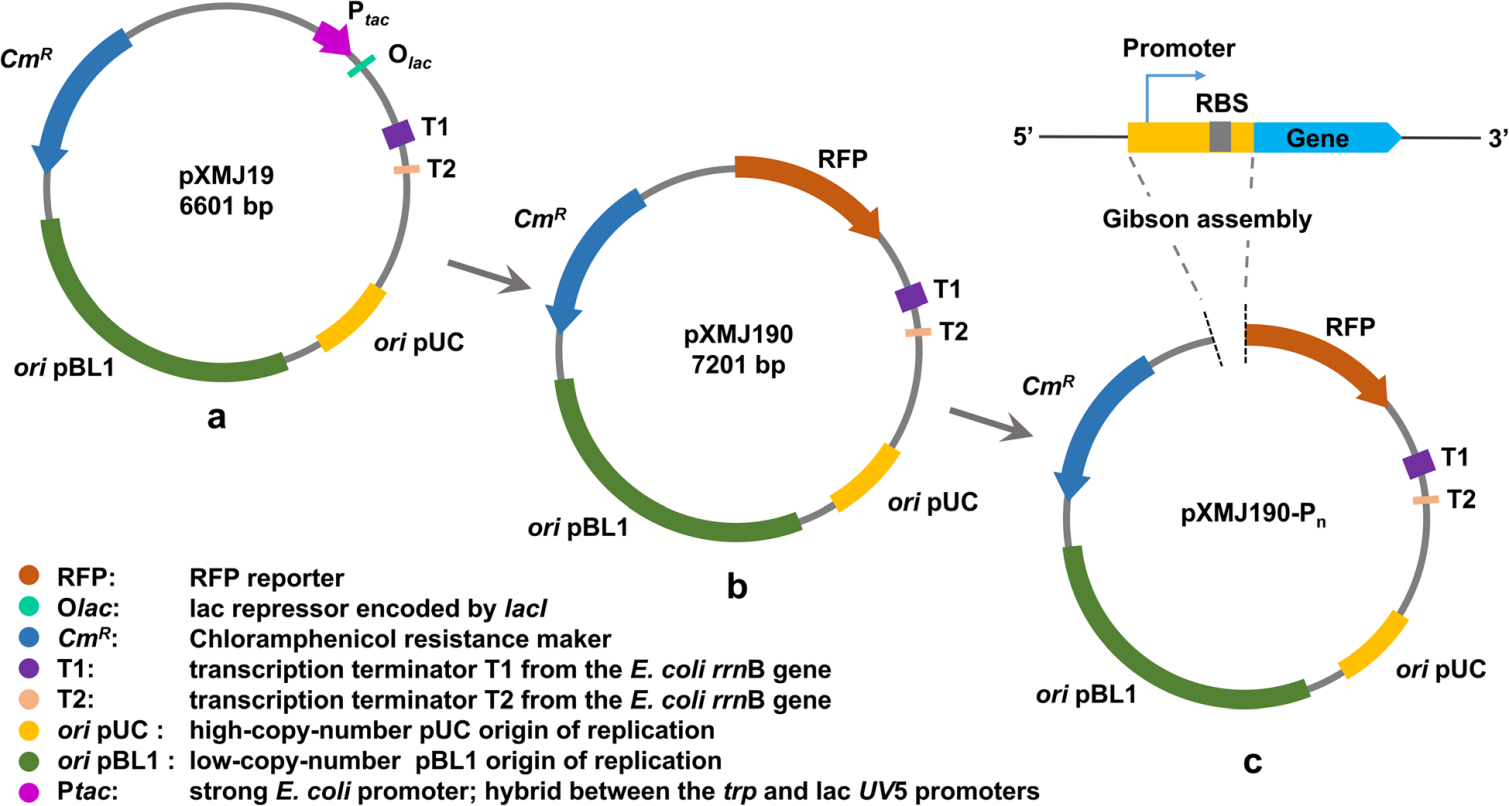
Flowchart for the construction of probe-vector that used for screening promoters. **a**: The shuttle vector pXMJ19 serves as the backbone. **b**: The probe-vector pXMJ190 with reporter of RFP and deletion of P_*tac*_ and O_*lac*_. **c**: pXMJ190-P_*n*_ represent vectors with the 26 putative promoters inserted in the upstream of RFP

For a comprehensive comparison, two conserved homologous promoters CJ1 and IJ59 that are active in *C. ammoniagenes* ATCC 6872 were aligned against the genome of *C. ammoniagenes* ATCC 6871 by BLAST and were cloned into pXMJ190 to give pXMJ190-CJ1 and pXMJ190-IJ59 [7, 15], respectively. Moreover, previous studies have shown promoters that work well in *C. glutamicum* may also be active in *C. ammoniagenes* [20]*.* Thus, a strong endogenous *C. glutamicum* promoter named P_*gro*_ (the promoter of *groES* gene) was cloned as pXMJ190-P_*gro*_ [19]. In addition, the most widely used *tac* promoter and conserved SD sequence was also selected and cloned into pXMJ190 as pXMJ190-P_*tac*_ [21]. The pXMJ190 was used as negative control. A flowchart for construction of the probe vectors to identify promoters is shown in Fig. 2. All of the above constructed plasmids were validated by sequencing and then transformed into *C. ammoniagenes* ATCC 6871.

### Analysis and comparison of promoters in C. ammoniagenes ATCC 6871

**Fig. 3:**
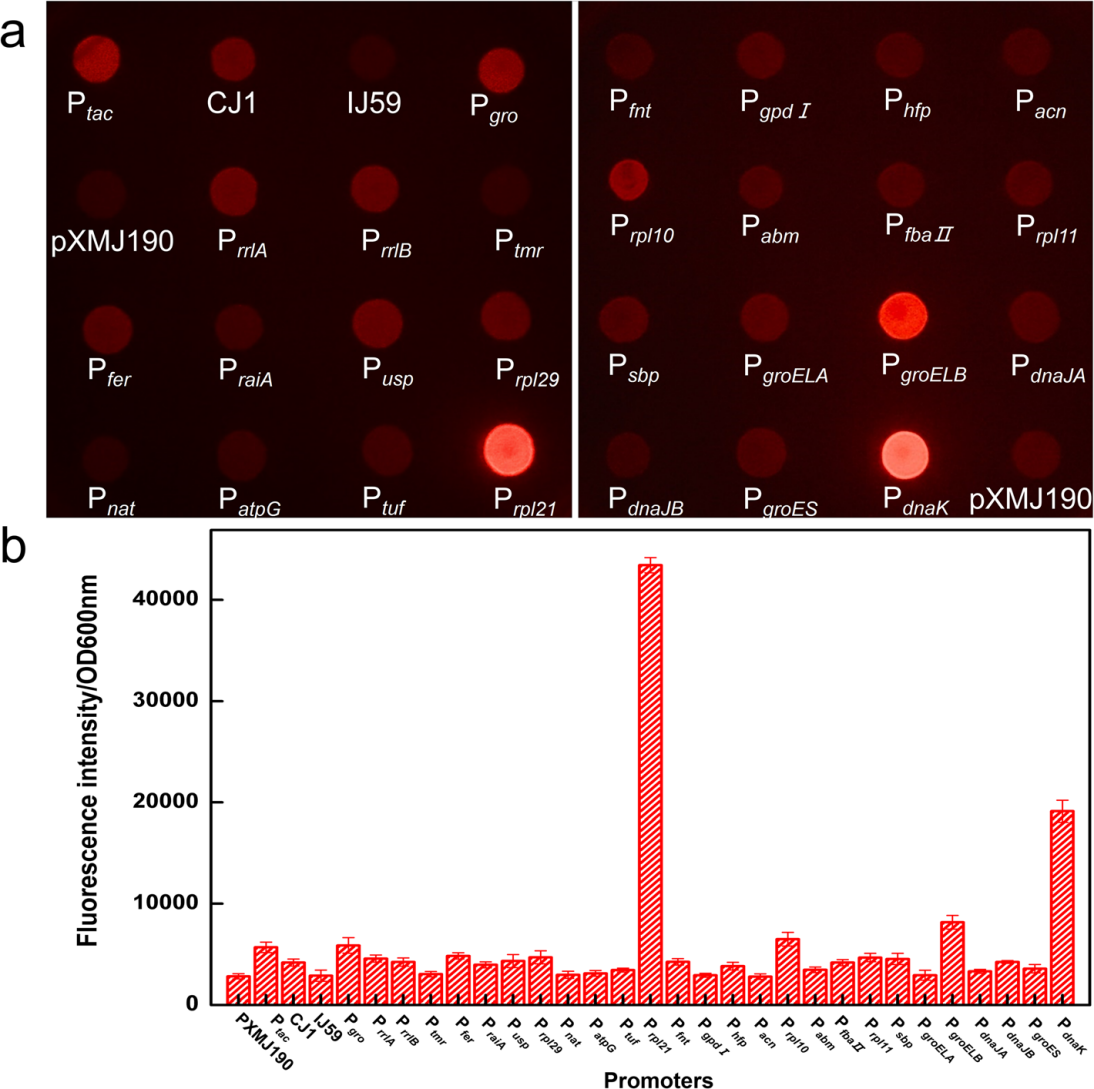
Different activities of selected promoters. **a**: Observed red fluorescence of bacteria using a LUYOR-3430 stereo microscope with a fluorescence excitation source (LUYOR, USA) set at 501 nm. **b**: Measured fluorescence intensities of bacteria using a Synergy H4 microplate reader. Cells were washed once with PBS (pH 7.4) and then resuspended in PBS (pH 7.4) at an OD_600 nm_ of approximately 1.0. The excitation wavelength for RFP was set at 554 nm and emission was set at 586 nm. Error bars show the standard deviation of three measurements

To analyze the activities of our cloned promoters, we first made a visual assessment of all *C. ammoniagenes* ATCC 6871 strains harboring pXMJ190, pXMJ190-CJ1, pXMJ190-IJ59, pXMJ190-P_*gro*_, pXMJ19-P_*tac*_ and pXMJ190-P_*n*_ series plasmids on LB plates containing 20 μg/ml chloramphenicol. Red fluorescence was observed by microscopy, with different intensities in different colonies, indicating that there was variable expression of RFP from the 26 putative promoters (Fig. 3a). Among these promoters, P_*rpl21*_, P_*rpl10*_, P_*groELB*_, and P_*dnaK*_ exhibited strong activity in *C. ammoniagenes* ATCC 6871, with P_*rpl21*_ being the strongest promoter. The remaining promoters had very low or non-existent red fluorescence.

To quantify the activities of the cloned promoters, growth-normalized fluorescence intensities were measured using a microplate assay and the data are shown in Fig. 3b and Additional file 1: Table S2, the results showed that we successfully isolated 20 endogenous promoters with different activities. Among the 20 promoters (P_*rrlA*_-P_*sbp*_) from genes with high transcription levels, P_*tmr*_, P_*nat*_, P_*atpG*_, P_*gpdI*_, P_*acn*_ are essentially inactive, while P_*groELA*_ from the 6 molecular chaperone promoters was also inactive. Meanwhile, four strong promoters, including the P_*rpl21*_ with 43,433 RFU/OD intensity and followed by P_*dnaK*_ (19,125 RFU/OD), P_*groELB*_ (8166 RFU/OD) and P_*rpl10*_ (6490 RFU/OD) were identified whose fluorescence levels were consistent with those observed on plates. Thus, a range of promoters with different transcriptional activities, including four strong promoters, were identified and can be used directly in further applications. According to the obtained data, only two promoters (P_*rpl21*_ and P_*rpl10*_) from the 20 genes with high levels of transcription had strong activities, while two promoters (P_*groELB*_ and P_*dnaK*_) sourced from the six molecular chaperone genes exhibited relatively high activities, which may indicate that isolating promoters from molecular chaperones may be a more efficient strategy. Although most of the promoters identified from the RNA-seq profiles exhibited low level expression or were silent, the red fluorescence intensity of P_*rpl21*_ was almost 2.3 times that of the highest promoter (P_*dnaK*_) derived from upstream regions of the molecular chaperones. This suggests isolating promoters from genes with high transcription levels may yield strong promoters.

When it comes to the known promoters, the promoter CJ1 had some certain activity (4171 RFU/OD), the IJ59 (2866 RFU/OD) promoter had almost non activity, and the activity of P_*gro*_ (5872 RFU/OD) was slightly higher than the P_*tac*_ (5683 RFU/OD) promoter but still lower than promoter P_*rpl10*_ (6490 RFU/OD). This demonstrates the promoters that work well in one species or homologous strains may not possess the same characteristics in another.

### Application of P_rpl21_ in C. ammoniagenes for improving the production of CoA

It has been reported that CoA, a ubiquitous and essential cofactor in biochemical reactions, can be produced by *C. ammoniagenes* ATCC 6871 [25]. In the CoA biosynthetic pathway, the committed step catalyzed by pantothenate kinase (coaA) is subject to feedback inhibition by CoA and acyl-CoAs [26]. Bacterial coaA proteins are categorized based on their amino acid sequences into three types, namely type I, II, and III. *C. ammoniagenes* carries a type I coaA which is highly regulated by CoA and its derivatives. In contrast, type II and III enzymes are insensitive to CoA and its thioesters [27, 28], therefore, in order to reduce feedback inhibition and increase the production of CoA, the type III pantothenate kinase from *Pseudomonas putida* (*Pp*coaA) was selected and overexpressed by the control of the strongest promoter P_*rpl21*_ in *C. ammoniagenes* ATCC 6871 [29, 30].

**Fig. 4:**
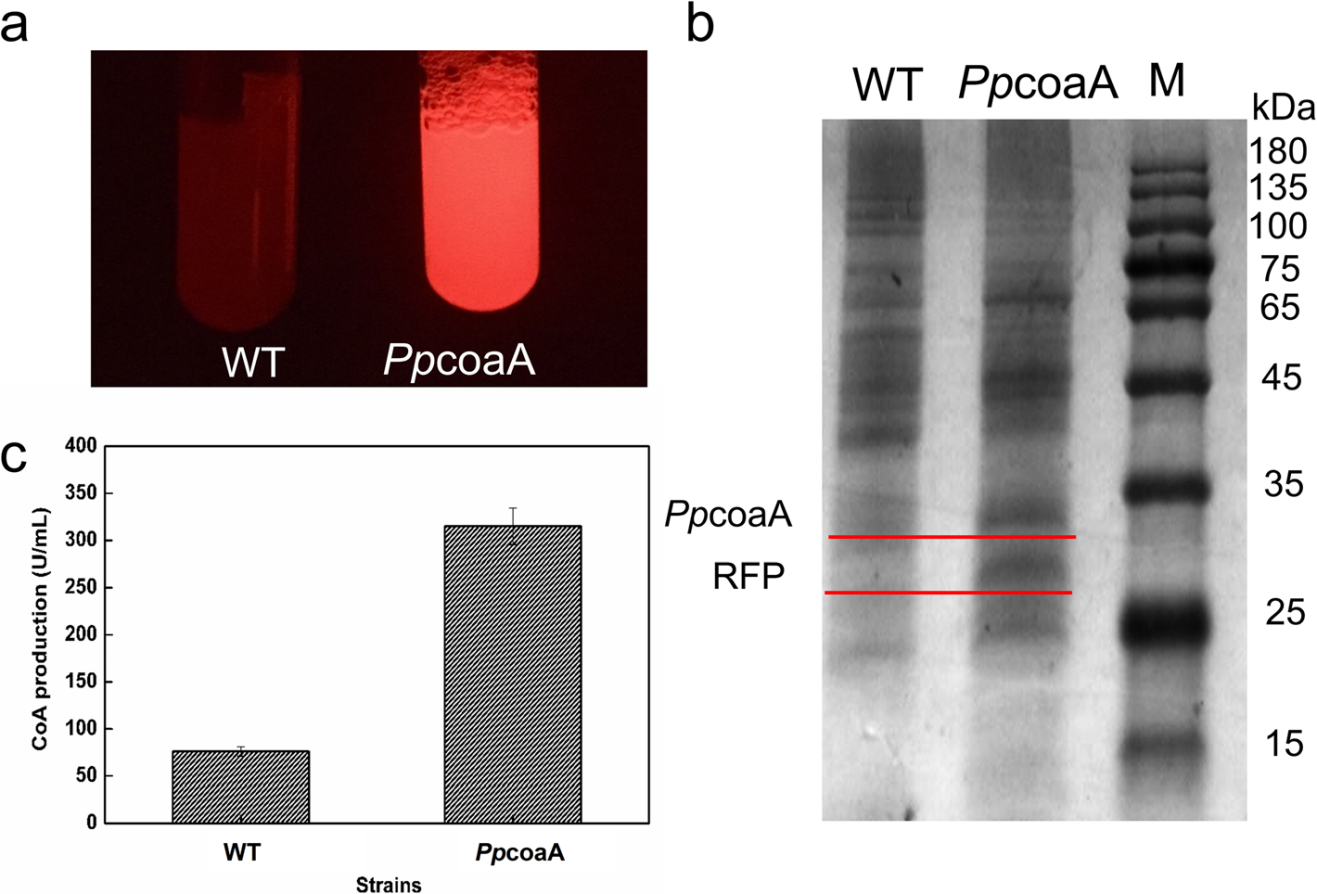
Application of P_*rpl21*_ in *C. ammoniagenes* for improving the production of CoA. **a**: The comparison of fluorescence intensities between cells harboring pXMJ190-P_*rpl21*_-*Pp*coaA-RFP and wild type cells. **b**: SDS–PAGE analysis of RFP and *Pp*coaA co-expression in *C. ammoniagenes*. Samples were prepared with an equal concentration of cells, and 40 μg of cell lysate were loaded per lane. Lane M: protein marker. **c**: The CoA production of cells harboring pXMJ190-P_*rpl21*_-*Pp*coaA-RFP and control. Error bars show the standard deviation of three measurements

Considering there are no direct enzyme assays for type III pantothenate kinase, RFP was co-expressed with *Pp*coaA. As shown in Fig. 4a, bright red fluorescence was observed in the tube with cells containing pXMJ190-P_*rpl21*_-*Pp*coaA-RFP, suggesting that RFP and *Pp*coaA were successfully co-expressed. By measuring the red fluorescence intensity, the strain harboring pXMJ190-P_*rpl21*_-*Pp*coaA-RFP had up to 7560 RFU/OD. This value was lower than that observed when RFP expressed alone under the control of P_*rpl21*_, possibly due to the long distance from the transcription initiation site and the increased cellular burden caused by co-expression of two proteins. The expression of RFP and *Pp*coaA was also be observed by SDS-PAGE. As shown in Fig. 4b, significant bands of approximately 27 kDa and 32 kDa were observed indicating that RFP and *Pp*coaA were overexpressed and that the P_*rpl21*_ promoter functioned well in *C. ammoniagenes*. Furthermore, three crucial substrates (pantothenic acid (2 mM), L-cysteine (2 mM), ATP (6 mM)) were added to the reaction mixture containing a certain number of bacterial cells (OD_600 nm_ ≈ 40) at 39 °C. Coenzyme A production increased to a satisfactory yield of approximately 315 U/mL in 6 h (Fig. 4c), which was almost 4.1 times higher than the same conditions without pXMJ190-P_*rpl21*_-*Pp*coaA-RFP in the cells (76 U/mL). The results indicated that *Pp*coaA was successfully overexpressed under the control of promoter P_*rpl21*_ and increased the anabolic flow of CoA. To further improve the CoA production, the RFP gene was removed from the plasmid pXMJ190-P_*rpl21*_-*Pp*coaA-RFP for eliminating the burden of RFP expression in cells. With the *Pp*coaA overexpressed alone in *C. ammoniagenes,* the CoA production was reached 332 ± 13 U/mL, which was slightly increased than the *Pp*coaA and RFP co-expressional system.

## Discussion

*C. ammoniagenes* ATCC 6871 is a producer of CoA, however, its genetic operation has been rarely reported, and its industrial application potential still needs to be improved. In order to further increase the production of CoA, endogenous promoters from *C. ammoniagenes* ATCC 6871 were screened in this study. Firstly, 20 putative promoters based on genes with high transcription levels and 6 promoters upstream of molecular chaperones were identified and characterized for transcriptional activity by using RFP as the reporter gene. Among the 26 putative promoters, 20 with different transcriptional activities were isolated including 15 from the high transcription level genes and 5 from molecular chaperone genes. The output efficiency demonstrates that isolating promoters from molecular chaperones (5/6) might be a more efficient strategy than using RNA-seq (15/20). Therefore, isolating promoters from molecular chaperone genes is more effortless and cost-effective than RNA-seq. These results may ascribe to that the RPKM values of the genes only represent the abundance of RNA and do not equivalent to its promoter activities, which might be caused by multiple factors, such as the codon usage of genes, copy numbers of genes, the half-lives of mRNA and limitations inherent in transcriptomics [22–24]. It thus, isolating promoters based on RNA-Seq requires further verification by experiments.

Nevertheless, we should note that the red fluorescence intensity of the highest promoter (P_*rpl21*_) identified from RNA-seq was almost 2.3 times than the highest promoter (P_*dnaK*_) derived from molecular chaperone genes, so isolating promoters based on the genes transcriptional data may be more likely to yield promoters with highest activity. To our best knowledge, P_*rpl21*_ from the upstream sequence of the 50S ribosomal protein L21 is the strongest *C. ammoniagenes* ATCC 6871 promoter reported so far. The promoters obtained in this work will enrich the available promoter toolkit for *C. ammoniagenes* and should be valuable in current platforms for metabolic engineering and synthetic biology for the optimization of pathways to extend the product spectrum or improve the productivity in *C. ammoniagenes* ATCC 6871.

To further verify the capacity of P_*rpl21*_, the key gene *Pp*coaA in the biosynthetic pathway of CoA was overexpressed in *C. ammoniagenes* ATCC 6871 with the aim of reducing the feedback inhibition and increasing the production of CoA. The CoA production of manipulated *C. ammoniagenes* was approximate 4.4 times to the control, which confirmed that *Pp*coaA overexpressed and functionalized successfully in *C. ammoniagene*. These results prove that the selected promoter for the overexpression of foreign genes in *C. ammoniagenes* could be used as an efficient tool for improving the yield of major products in *C. ammoniagenes*. However, eliminating the burden of co-expressing RFP only slightly increase the CoA production, possibly due to the undiscovered limitations exist in the CoA synthetic pathway. Thus, more efforts should be focus on the resolving of rate-limiting steps of CoA synthesis in *C. ammoniagenes*.

## Conclusions

In summary, this study provides a rational strategy to isolate endogenous promoters from *C. ammoniagenes* ATCC 6871, which may be helpful in other similar scientific research. Through this strategy, we successfully isolated a range of promoters with different transcriptional activities and the strongest one was applied to improve the CoA production in *C. ammoniagenes* ATCC 6871, raising hope for further improving the industrial production level of CoA.

## Methods

### Bacterial strains, media, and growth conditions

The *C. ammoniagenes* ATCC 6871 was purchased from China General Microbiological Culture Collection Center (CGMCC, Beijing, China). *E. coli* Top10 (Tsingke, Beijing, China) was cultivated in Luria-Bertani (LB) broth or on LB plates with 2% (w/v) agar at 37 °C as a host for transformation. *C. ammoniagenes* ATCC 6871 grown in NCM medium at 30 °C was used as the transformation host for *E. coli/*Corynebacteria vectors used in this study. After transformation by electroporation (1.8 kV), transformants were plated on BHIS plates with 20 μg/mL chloramphenicol at 30 °C. Fermentation medium (glucose 100 g/L, peptone 12 g/L, yeast powder 8 g/L, MgSO_4_·7H_2_O 10 g/L, KH_2_PO_4_ 10 g/L, K_2_HPO_4_ 10 g/L, urea 7 g/L, pH 7.5–7.8) was used to cultivate strains producing CoA. Cultivation for expression analysis was performed in at least biological duplicates.

### Recombinant DNA techniques

*C. ammoniagenes* genomic DNA was isolated using a genomic DNA isolation kit (Tiangen, Beijing, China). Kits for plasmid isolation, extraction of DNA from agarose gels and PCR product purification were also purchased from Tiangen (Beijing, China). I-5 2 × High-Fidelity Master Mix and Trelief™ SoSoo Cloning Kit were purchased from Tsingke (Beijing, China) and used for routine molecular biology applications.

### Construction of probe-vector pXMJ190

The shuttle vector pXMJ19 (a kind gift from Professor Dong, Fig. 2a) was used as the backbone for vectors constructed in this study. To eliminate interference from the promoter already present in pXMJ19, the *tac* promoter and *lac* operator were removed. A gene encoding red fluorescent protein (RFP) was inserted into the MCS to form a probe-vector named pXMJ190 (Fig. 2b). Gibson assembly was used to assemble DNA fragments upstream of the reporter gene *rfp*, resulting in seamless ligation between fragments and the probe-vector [31, 32].

### Genetic manipulation of C. ammoniagenes

To increase transformation efficiency, recipients were grown in 100 mL of NCM medium at 30 °C until an OD_600 nm_ of approximately 1.0. Cells were incubated on ice for 20 min and harvested by centrifugation in a polypropylene tube at 4000 rpm for 10 min at 4 °C. After washing twice in cold distilled water and two washes in ice-cold 10% glycerol, cells were resuspended in 1 mL of 10% glycerol. For electroporation, 100 μL of the freshly prepared electro-competent cells were mixed with 3 μL plasmid (50 ng/μL in ddH_2_O) in a cold sterile electroporation cuvette (1 mm electrode gap) and pulsed immediately with a MicroPulser electroporator (Bio-Rad Laboratories, Inc., Hercules, CA). The electroporator was usually set at a voltage of 1.8 kV. Cells were subsequently resuspended in 0.9 mL of BHIS, heated at 46 °C for 6 min and withdrawn immediately for recovery by incubating for 3 h at 30 °C and then plated for selection of transformants.

### Construction and analysis of selected promoters

The transcriptional levels of genes can be estimated by Reads Per Kilobase per Million mapped reads (RPKM), so the genes were ranked by RPKM value based on transcriptional data from *C. ammoniagenes* and the top 20 genes were selected. All of the genes were identified from the genome and their promoters were predicted as below. Six annotated molecular chaperones in the genome were sorted and their promoters were predicted as outlined below. According to previous studies, the promoter-5′-UTR junction influences mRNA and protein levels [23, 33]. Therefore, we integrated the corresponding 5′-UTR into each promoter.

Promoter sequences were predicted using the Neural Network Promoter Prediction online tool (http://www.fruitfly.org/seq_tools/promoter.html) [34]. The promoter prediction score threshold was set to 0.7. Moreover, due to the possibility of tandem promoters, all eligible promoter sequences within 300 bp upstream of the start codon were considered [35]. For correct transcription initiation, the complete “promoter” consisted of 5′-UTR, predicted promoter region and Shine-Dalgarno sequence, which typically correspond to the region 60 bp upstream of a predicted promoter and 1 bp upstream of the start codon. The 60 bp extension upstream of the promoter accounts for potential UP-elements [36, 37].

Promoters were amplified from *C. ammoniagenes* ATCC 6871 and *C. glutamicum* ATCC 13032 genomic DNA with the corresponding primers. Oligonucleotide primers used in this work are listed in Additional file 1: Table S1. PCR products were ligated into the vector pXMJ190 using Gibson assembly as shown in Fig. 2c. All plasmids were constructed in *E. coli* TOP10 and then transformed into *C. ammoniagenes* ATCC 6871 for subsequent analysis.

### Fluorescence intensity assay

To evaluate RFP expression under the control of the various promoters, *C. ammoniagenes* strains harboring various vectors were grown overnight on LB plates containing 20 μg/ml chloramphenicol. Fluorescence was observed using a LUYOR-3430 stereo microscope with a fluorescence excitation source (LUYOR, USA) set at 501 nm and matching lenses to detect RFP. Pictures were captured with a camera.

For more accurate comparisons, the fluorescence intensities of bacteria harboring different plasmids were measured using a Synergy H4 microplate reader (BioTek, USA). In order to exclude other interfering factors, harvested cells were washed once with PBS (pH 7.4) and then resuspended in PBS (pH 7.4) at an OD_600 nm_ of approximately 1.0. The excitation wavelength for RFP was set at 554 nm and emission was set at 586 nm. Fluorescence intensities were normalized by OD_600 nm_ and were used to indicate the activities of the promoters. Bacteria harboring pXMJ19-P_*tac*_ were induced with 1 mM IPTG.

### Construction of the recombinant plasmid pXMJ190-P_rpl21_-PpcoaA-RFP

To further examine the function of the isolated promoters, a type III pantothenate kinase from *P. putida* KT2440 (*Pp*coA) and RFP were co-expressed under the control of the strongest promoter P_*rpl21*_. The *PpcoaA* gene was amplified by PCR from the genomic DNA of *P. putida* KT 2440, and the Shine-Dalgarno sequence for translation of RFP was calculated using the RBS calculator online tool (https://www.denovodna.com/software/). All primers used in this section are listed in Additional file 1: Table S1. The recombinant plasmid pXMJ190-P_*rpl21*_-*Pp*coaA-RFP was constructed with Gibson assembly and positive colonies were confirmed by DNA sequencing (Tsingke, China). Recombinant plasmids were transformed into *C. ammoniagenes* ATCC 6871 for further experiments.

### SDS-PAGE analysis

*C. ammoniagenes* strains were precultured in 10 mL LB medium at 30 °C and shaken at 220 rpm for 24 h. Ten percent of the culture was inoculated in a 250 mL shake flask containing 100 mL fermentation medium. After 24 h cultivation, cell samples were harvested by centrifugation at 12,000 rpm for 10 min. Forty micrograms of cell lysate were loaded per lane. The *Pp*coaA and RFP expression was analyzed by 15% (v/w) polyacrylamide gel electrophoresis (PAGE) with cell-free extract under denaturing conditions. Mini-Protean III Electrophoresis System (Bio-Rad, USA) was utilized to perform the operation. Coomassie Brilliant Blue R-250 (0.2%, w/v) was utilized to stain protein on the gel.

### Analysis of CoA production

Coenzyme A content was determined according to the modified phosphotransacetylase method [38, 39]. All reagents were purchased from National Institutes for Food and Drug Control. Briefly, 3.0 mL of Tris buffer (pH 7.6), 0.1 mL of acetyl phosphate dilithium salt (15.2 g/L) and 0.1 mL of the test solution were added into a 1 cm cuvette and mixed. Absorbance at 233 nm was recorded as E_0_; and then 0.01 mL of the phosphotransacetylase (30–40 U/mL) solution was added, mixed well and the highest absorbance within 3 to 5 min was taken as E_1._ Finally, another 0.01 mL of phosphotransacetylase solution was added, mixed well and the absorbance was read as E_2_. The number of CoA units per milliliter was calculated as U = (2E_1_-E_0_-E_2_) × 5.55 × 413.

## Supplementary information

**Additional file 1: Table S1.** Primer sequences used in this study. **Table S2.** Comparison of different promoter activities in *C. ammoniagenes* ATCC 6871. **Table S3.** The sequences of identified promoters.

## Data Availability

All data generated or analyzed during this study are included in this manuscript and the Additional files associated with it.

## Abbreviations

CoA: coenzyme A
IPTG: Isopropyl β-D-1-thiogalactopyranoside
