# Supplementary material for: Isolating promoters from Corynebacterium ammoniagenes ATCC 6871 and application in CoA synthesis
Source: BMC Biotechnol. 2019 Nov 12;19:76. doi: 10.1186/s12896-019-0568-9 (PMC6849255; doi:10.1186/s12896-019-0568-9)
Supplement: Supplementary file 1 — Additional file 1: Table S1. Primer sequences used in this study. Table S2. Comparison of different promoter activities in C. ammoniagenes ATCC 6871. Table S3. The sequences of identified promoters. [file 12896_2019_568_MOESM1_ESM.docx]

**Supplementary materials**

**Isolating promoters from** ***Corynebacterium ammoniagenes* ATCC 6871 and application in CoA synthesis**

Yingshuo Hou^1,2^·Siyu Chen^1^·Jianjun Wang^1^·Guizhen Liu^3^·Sheng Wu^1^·Yong Tao^1^

^1^ CAS Key Laboratory of Microbial Physiological and Metabolic Engineering, Institute of Microbiology, Chinese Academy of Sciences, Beijing 100101, People’s Republic of China

^2^ University of Chinese Academy of Sciences, Beijing 100101, People’s Republic of China

^3^ Kaiping Genuine Biochemical Pharmaceutical Co. Ltd

Correspondence: Sheng Wu, Yong Tao, CAS Key Laboratory of Microbial Physiological and Metabolic Engineering, Institute of Microbiology, Chinese Academy of Sciences, Beijing 100101, People’s Republic of China. Tel. 86-10-64807417. E-mail: [shengwu@im.ac.cn, taoyong@im.ac.cn](mailto:shengwu@im.ac.cn,%20taoyong@im.ac.cn).

Table S1. Primer sequences used in this study.

| Primer | Sequence(5’-3’) |
| --- | --- |
| P*_rrlA_* F | ATATTCTGAAATGAGCTGgcatcacgaaacacatacta |
| P*_rrlA_* R | GTCCTCGGAGGAGGCCATacaccccacacaccacaaag |
| P*_rrlB_* F | ATATTCTGAAATGAGCTGgcatcacgaaacacatacta |
| P*_rrlB_* R | GTCCTCGGAGGAGGCCATacaccccacaaccaacacac |
| P*_tmr_* F | ATATTCTGAAATGAGCTGccgtcgcatcaaaggcatca |
| P*_tmr_* R | GTCCTCGGAGGAGGCCATattgatgtaggcgggatgtg |
| P*_fer_* F | ATATTCTGAAATGAGCTGtccgcggtctttgccgatct |
| P*_fer_* R | GTCCTCGGAGGAGGCCATgattttcctcttccttcgta |
| P*_raiA_* F | ATATTCTGAAATGAGCTGcacgcttgctgccacggtcg |
| P*_raiA_* R | GTCCTCGGAGGAGGCCATgacttgcctccctgtgatcg |
| P*_usp_* F | ATATTCTGAAATGAGCTGcaccatcttgtacccatcaa |
| P*_usp_* R | GTCCTCGGAGGAGGCCATgatgttcccttcctcatttt |
| P*_rpl29_* F | ATATTCTGAAATGAGCTGgactacaccatccccttccg |
| P*_rpl29_* R | GTCCTCGGAGGAGGCCATgcttatcctcttcctcgctt |
| P*_nat_* F | ATATTCTGAAATGAGCTGctgctaaatacttgcgggga |
| P*_nat_* R | GTCCTCGGAGGAGGCCAT ggttcccagtctacacttga |
| P*_atpG_* F | ATATTCTGAAATGAGCTGtggcgctgattttgttattt |
| P*_atpG_* R | GTCCTCGGAGGAGGCCATggccaatgttgtaacgctca |
| P*_tuf_* F | ATATTCTGAAATGAGCTGaaccacgttcaggcggctac |
| P*_tuf_* R | GTCCTCGGAGGAGGCCATtgtatgtcctcctggacttc |
| P*_rpl21_* F | ATATTCTGAAATGAGCTGagggccgttttctagcccgc |
| P*_rpl21_* R | GTCCTCGGAGGAGGCCATagagggttaccccttatcta |
| P*_fnt_* F | ATATTCTGAAATGAGCTGgttaacagtacgaaagtact |
| P*_fnt_* R | GTCCTCGGAGGAGGCCATgcatcctcacttgaaaagaa |
| P*_gpdⅠ_* F | ATATTCTGAAATGAGCTGaaatagaccagaatttcggg |
| P*_gpdⅠ_* R | GTCCTCGGAGGAGGCCATggtagttgtctcctaaaaag |
| P*_hfp_* F | ATATTCTGAAATGAGCTGgacgctgcgagcccagtttc |
| P*_hfp_* R | GTCCTCGGAGGAGGCCAT gaaactcctccaaaagtcgg |
| P*_acn_* F | ATATTCTGAAATGAGCTGgtcggctacgtcaattccct |
| P*_acn_* R | GTCCTCGGAGGAGGCCATtccttgttgttttgaactaa |
| P*_rpl10_* F | ATATTCTGAAATGAGCTGtacgcccgctcgctacgttc |
| P*_rpl10_* R | GTCCTCGGAGGAGGCCATtttcttactcgcctcctttc |
| P*_abm_* F | ATATTCTGAAATGAGCTGtgcagctaccagatgccggc |
| P*_abm_* R | GTCCTCGGAGGAGGCCATggcctccatcctacccaaaa |
| P*_fbaⅡ_* F | ATATTCTGAAATGAGCTGgctgctggaagccgccgctc |
| P*_fbaⅡ_* R | GTCCTCGGAGGAGGCCATgaattcttccttcttacgcg |
| P*_rpl11_* F | ATATTCTGAAATGAGCTGgcaagatggttggcctagtc |
| P*_rpl11_* R | GTCCTCGGAGGAGGCCATcgaattacctctttccgtga |
| P*_sbp_* F | ATATTCTGAAATGAGCTGcttgaggtagtcctgcgtgc |
| P*_sbp_* R | GTCCTCGGAGGAGGCCATttccatccttgaatgtttaa |
| P*_groELA_* F | ATATTCTGAAATGAGCTGGcacaaacgcaactaagaga |
| P*_groELA_* R | GTCCTCGGAGGAGGCCATgtgtggctaaaccgccttac |
| P*_groELB_* F | ATATTCTGAAATGAGCTGGcggatgggcggatttatgc |
| P*_groELB_* R | GTCCTCGGAGGAGGCCATagtgttttaatctcctagat |
| P*_dnaJA_* F | ATATTCTGAAATGAGCTGCgtggtaggtcccacattca |
| P*_dnaJA_* R | GTCCTCGGAGGAGGCCATggggagatgtaattcctttt |
| P*_dnaJB_* F | ATATTCTGAAATGAGCTGCaatgccatggttattatcg |
| P*_dnaJB_* R | GTCCTCGGAGGAGGCCATtgggcatctcctcctttcat |
| P*_groES_* F | ATATTCTGAAATGAGCTGCtatgcttggctagcaggca |
| P*_groES_* R | GTCCTCGGAGGAGGCCATggtgatgattcctccgtgtg |
| P*_dnaK_* F | ATATTCTGAAATGAGCTGTaactcaaaaaaatgggcat |
| P*_dnaK_* R | GTCCTCGGAGGAGGCCATatttatgttctccttctagc |
| J190 F | ATGGCCTCCTCCGAGGACGTCATCA |
| J190 R | CAGCTCATTTCAGAATATTTGCCAG |
| *Pp*coaA F | gataaggggtaaccctctAtgattcttgagctcgattg |
| *Pp*coaA R | AAACGTGTGTAAATGTTGAATCGGGGTGTATTGTGAAtcaatccaatggacaagccattgccaggccaacgaaaacc |
| pXMJ190- P*_rpl21_*-RFP F | TCAACATTTACACACGTTTTTCTCCTTAGATTTTTTTATGGCCTCCTCCGAGGACGTCATCAAGGAGTTCATGCGCT |
| pXMJ190- P*_rpl21_*-RFP R | agagggttaccccttatctaaactcggctc |

The red letter indicates the Shine-Dalgarno sequence and the underlined sequence is the overlap region.

Table S2. Comparison of different promoter activities in *C. ammoniagenes* ATCC 6871.

| Name | Average fluorescence intensity (RFU/OD) |
| --- | --- |
| pXMJ190 | 2801 |
| P*_tac_* | 5683 |
| CJ1 | 4171 |
| IJ59 | 2866 |
| P*_gro_* | 5872 |
| P*_rrlA_* | 4564 |
| P*_rrlB_* | 4224 |
| P*_tmr_* | 3014 |
| P*_fer_* | 4835 |
| P*_raiA_* | 3937 |
| P*_usp_* | 4313 |
| P*_rpl29_* | 4694 |
| P*_nat_* | 2949 |
| P*_atpG_* | 3089 |
| P*_tuf_* | 3420 |
| P*_rpl21_* | 43433 |
| P*_fnt_* | 4240 |
| P*_gpdⅠ_* | 2905 |
| P*_hfp_* | 3812 |
| P*_acn_* | 2765 |
| P*_rpl10_* | 6490 |
| P*_abm_* | 3450 |
| P*_fbaⅡ_* | 4159 |
| P*_rpl11_* | 4668 |
| P*_sbp_* | 4531 |
| P*_groELA_* | 2908 |
| P*_groELB_* | 8166 |
| P*_dnaJA_* | 3289 |
| P*_dnaJB_* | 4222 |
| P*_groES_* | 3556 |
| P*_dnaK_* | 19125 |

Table S3. The sequences of identified promoters.

| Promoter | Sequence |
| --- | --- |
| P*_rrlA_* | Gcatcacgaaacacatactaaagactctttgctgagtgggtgtgtggttggtggtgtggggtgttgtgtgagaactgtatagtggacgcgagcatccaccacacgagtgcttttgaagtgtttgtgtggtgtgtgtaatttttcttattttttctgtgagaacgtcgcataccaactttggttggtgtgtgtttagtattttttagttatggttgttcacccccatgcactttgtggtgtgtggggtgt |
| P*_rrlB_* | gcatcacgaaacacatactaaagactctttgttgagtgggtgtgtggttggtggtgtggggtgttgtgtgagaactgtatagtggacgcgagcatccaccacatgaatatgctttgtgtgtgtttgtgtggtgtgtgtaatttttcttattttttctgtgagaacgtcacatgccagtctttgttggttggtgtgtggtttagtgttttttagttatggttgttcacccccacagctgattgtgtgttggttgtggggtgt |
| P*_tmr_* | ccgtcgcatcaaaggcatcaatgcctaacctctgagccgacgaaagccaaaagcttgttcgtcgcttgtgggaattaatcgcgctgttgtagtgttgttaagaatgtaggaagatgcacatcccgcctacatcaat |
| P*_fer_* | tccgcggtctttgccgatctcgcgcaacgccactgcgccctataaatgaggtagtcattggtttgatggctgagctatgaagtaaaagctatcaaactgtgatgattaaaaaccgtggctgttctgcgtgtttaagcttgtaatcttagggtttccaaattttgggtgggcttacctacgtggcacaggctgcgcaagccctctaatgttgtaagcgaacgcagcggacagaagctgcgcggtaggtaccgaaggctaacggttttctgtgcacgcttttagtacctgcaacactcgcattttgaagagtatgacgtagaagtacgaaggaagaggaaaatc |
| P*_raiA_* | cacgcttgctgccacggtcgaagtcttgcgcggggcgggggcaaatgtcgccggaggattagtttttgccgatgtttaaggacattggttatgaccactggtagaatagggattgtcacacacagtagttgttaatatctacgtcacatctactgcttgtgagtacatccggccccaggggcccaaggtttggccccgcgatcacagggaggcaagtc |
| P*_usp_* | caccatcttgtacccatcaaatgcggaccccgcgacaatacttcctcaggtcgacccatctgctgcgattcaaacagcggtggagaacctataattccgaaagttttctcgggctgcccactgattagggtataacaacctatccaagtggtaataaacagctatacttaagtgtaggaaatgaaatcatttcatccccgtagaatttcacccgaccagtgcgggattgccgttaactggctgaaaatgaggaagggaacatc |
| P*_rpl29_* | gactacaccatccccttccggattttggctcactatgaaccagtactgtgcctgcgcgtttgccaggttgcagtcgctgttgtgtaagtcagaatcaaactggcgttggcaccaggctctttgcccggacaacgttatagagaacatcgagaagcaatttcccaccgcttcgcgccccggatacgccgggaatgtgaaagtggggaagcgaggaagaggataagc |
| P*_nat_* | ctgctaaatacttgcggggagaagctcgggcaaaggtcggctaaactggcgcttgaactgcggatttgtattgctgagcgcgcgttatgtaaagtaacatatcgtgcctaaggggcacagataaattaaataaggccctgtggcgcagttggttagcgcgccgccctgtcacggcggaggtcgcgggttcaagtcccgtcagggtcgcaagattgtccttgcggcaatctttggccagatagctcagtcggtagagcacacgcctgaaaagtgtggggtcgccagttcgatcctggctctggccacaacataaagctcctagtgtgaaaacattaggagctttgttgtttctcaagtgtagactgggaacc |
| P*_atpG_* | Tggcgctgattttgttatttgaaggcggggttaggttgggggtcggggtttccttgacctgcggatcgaaataacccgtgatgagctgatattatttcctgcgggttaccgcggaagcggcattagtaagattcgtgtcctgcgaaaatttatgccgtatcccatttacggtgattccctgttacaacttcgctgatgtgcgacggagtccgtagttgtgacagagagtttgagacgtccatcgcaccgtacgaccacgacaatcgtacggcccgaacacgggagagaacgctgagcgttacaacattggcc |
| P*_tuf_* | aaccacgttcaggcggctaccccaggagggtaggggtcacgctgaatcaataggtacaatcttgttgattgagctggccgttaccctcctaggatccgagatgattcttgtcgaggactaacgtccgcacaaatcttccgcggacaacctcaaaccccttttagctggtttgaaaattccgtggcataaatctaggatcgtgtaactggcacgaaaagaaagcgtcatgggcgcttggtattatcttccgggatattctaagtgctgtgacaactgtcaatcacgtggctgcgaaagtcgtagtcacaatgaagtccaggaggacataca |
| P*_rpl21_* | agggccgttttctagcccgctgggctttacgtggccggttgtatgagagtgcagccggccacgattttcggatctgcggaagcgggcggtactcttacttttaggcaagatgtactcattgtgagtattggctgcgcaacagtcaaagtggatcgaggccgtgtcgatgacgcggtttgtattttaggcctctctcggggtactctttgacagttgctgtttaggcgaagattttgccgtcctgttgggatggtgcaccgcctaggcactgtttgaagtaattgtccattcagggacttgtcttattagctaacgcccaggcaggttcttgagccgagtttagataaggggtaaccctct |
| P*_fnt_* | gttaacagtacgaaagtactgttattgcactgcgcaaacgctagctgcatgaacactttccgcaggtgaattaggcaatattagtgttgtttaattaaacgggttttatcgttttaaaccaataaatattgagcatagcaagctaatctagtccttaggtacccacaagttcacctttcttttcaagtgaggatgc |
| P*_gpdⅠ_* | aaatagaccagaatttcgggcatgaaggccctattgcgtgccttaaagctccccacgccagatattgagacataaatcacgtccgcaaatatgtctaatcgggcatattcggacacatatgggctgaccttggcaaaccgtagcccattttgtcgatttctcccgccgaaaaagtttgttaatgttggaatatccgaggaatgtgtgggtttgcttttataaatctgcgcagtgtatgcaacctcggtactatcggcagtgtcggagaaaaacatcatagaatttacgtctaagacgaattctcctaggcaattgcttttgtcgtactccgctttcccaactttttaggagacaactacc |
| P*_hfp_* | gacgctgcgagcccagtttcctagtgctctcccccattcccgggggcaagcctggtcaatattttcctgcagacggcattttattcggcgatattcctgagattgaatgcccaaggaaaccacggcgaaagccctgcgcccatgcacaatctcttggctagactacccctccgagaaacttcacatgtttagcaatgggggcaaatatgaccaagcccatattgaagaattaataaacacggataaagtcgtacttaatcatttgtttccgacttttggaggagtttc |
| P*_acn_* | gtcggctacgtcaattccctccgggaccatggaacctaccccttttgtcgtgtagtccgtgtgttgatatttctagctacccctctggttatatccaagtggggcctgcggagctgcgtcgacgcgccgaggaatgctaaagtctggaaacagtacgagcgttctgttacaatgaggggcgttcgactcgatacctcactatgatgggtggctgaaccatttggttagcctcattgacgtttggcttagaaccccatgactcaagcggtttgaaggcagcgagtggtgaggaaaaccgaatgtttagttcaaaacaacaagga |
| P*_rpl10_* | tacgcccgctcgctacgttcagtggtgggcaacgcggcgctatgtacggggcatggcaccgtgatttggaaaatgtggtagtagcgcgttaaggtaagtcccgaagtttgaacgagcctttctggttcgcgctcaagtttcaccgaagaccgtaggttatctgaaatccgttcagatcgaaggtccccgcactcatcggggcagcccacgcaggagacacttgttgcactctaagtctgaaaaagacttgtttgtgtggcctcgtgctcttgcacggggcttttttgatgcctgtcatcaaatttgctccggcggaacaaaagagatgttttgaaaggaggcgagtaagaaa |
| P *_abm_* | tgcagctaccagatgccggctttcacaccttaaagatggtgcggggagccggtatttttatttcccatttgcacgctggggaggcacgcgaaattttgggtaggatggaggcc |
| P*_fbaⅡ_* | gctgctggaagccgccgctcgcgtagcgaaatctttagaacgcagttaatctcacaggcgagttggcggcgggaaatgcttcacacaggcatactagttagtgacatttcattccacgcgtaagaaggaagaattc |
| P *_rpl11_* | gcaagatggttggcctagtctccatcttcggccgtgagaccccagtggagctttccccaaccgagattgagcgcatcaattaatttgggtaataccccgcctctagattaggctaggtcgacgtgtgtaaatttgcttgcacacgtcgattttttcatttactccccggtggccgcacaaggtggcatccggacgggccctgctattcatcgtggtgggataacccggtatcacggaaagaggtaattcg |
| P*_sbp_* | cttgaggtagtcctgcgtgcgctccggcaaatctgtcacatgcatacccccatcctaaacaaaccgttttaaaaacaaatcttcatccgtatgctttcaattgttcaacccattgaacttaaacattcaaggatggaa |
| P *_groELA_* | Gcacaaacgcaactaagagatttcacacggaggaatcatcaccatggcaaacattaagccacttgaggacaaggttcttgttcagattgtagaagctgaaaccaccaccgcatccggtctggttatcccagattctgccaaggaaaagccacaggaagcaaccgttgttgcagtcggcccaggccgcgcaaacgacaagggtgagctgaccccagtcggcgtcaacgaaggcgacaccgtcatcttctccaagtacggcggaactgagctcaagtacgacggcgaagagttcttgttgctgtctgctcgtgacctgctcgctgtaatcgagaagtaagtaaggcggtttagccacac |
| P *_groELB_* | Gcggatgggcggatttatgcccgtgcaggtgagccggatttttcggatcgcccgtggcgttttttcttgcactcgaggggtgagagtgccagaatggttgctgacaccaggttgaggttggtacacactcaccaatcctgccgtcgcgggcgcctgcgtgaccgataacccttgagtgaaacccaatctaggagattaaaacact |
| P*_dnaJA_* | Cgtggtaggtcccacattcatggactattccggaacaatgtctcgggtctcagccgttgcacattatgtcagcgatattctcggacatgaataattggcactcacctgtagagtgtcttaggcacaaatttaagttttaattaaaagatagcaacgaaaaggaattacatctcccc |
| P*_dnaJB_* | Caatgccatggttattatcgctgatgctgattcagaggactcagctcagtctgaaagctagccagcgtgaaagggggcgtcaggggtgttattacaccaactctgacgcccttcctgttgttagtggcagtattaagaattaaataattagcttagaaaaaacaataattacttatgacaattttcaatcttctatgaaaggaggagatgccca |
| P *_groES_* | Ctatgcttggctagcaggcaagaagcggcgttcgcgcacagctaaccgccgatgtgaaggcttttgttgagagctggcacttgcgcgggtagagtgctaatcaaggttgtttgacccacagttgttcacccgcgacgacggctgtgctggactaatgatgcaaccggcacaaacgcaactaagagatttcacacggaggaatcatcacc |
| P *_dnaK_* | Taactcaaaaaaatgggcatctgtaagcttcctccaattgtatagaaaaacctgaaggcgtgtcttttgaaggggtgtttaggattttctaaaaacatttttacccagtttacctgcggatatagtgggctttaaaagaaagttgagtcaggcgggaacaactttggaacttgtgccgttatactagttgagtgaccaacactcaggatggctaacaaagtgcacaactttctgttagtgtgggcgagtagctcattcaaagttgagtcacgtacaatcaacttaaatgaaacccaagctagaaggagaacataaat |
